# Modeling and Simulation of Turbulent Flows through a Solar Air Heater Having Square-Sectioned Transverse Rib Roughness on the Absorber Plate

**DOI:** 10.1155/2013/827131

**Published:** 2013-10-09

**Authors:** Anil Singh Yadav, J. L. Bhagoria

**Affiliations:** ^1^Mechanical Engineering Department, Technocrats Institute of Technology-Excellence, Bhopal 462021, India; ^2^Mechanical Engineering Department, Maulana Azad National Institute of Technology, Bhopal 462051, India

## Abstract

Solar air heater is a type of heat exchanger which transforms solar radiation into heat energy. The thermal performance of conventional solar air heater has been found to be poor because of the low convective heat transfer coefficient from the absorber plate to the air. Use of artificial roughness on a surface is an effective technique to enhance the rate of heat transfer. A CFD-based investigation of turbulent flow through a solar air heater roughened with square-sectioned transverse rib roughness has been performed. Three different values of rib-pitch (*P*) and rib-height (*e*) have been taken such that the relative roughness pitch (*P*/*e* = 14.29) remains constant. The relative roughness height, *e*/*D*, varies from 0.021 to 0.06, and the Reynolds number, Re, varies from 3800 to 18,000. The results predicted by CFD show that the average heat transfer, average flow friction, and thermohydraulic performance parameter are strongly dependent on the relative roughness height. A maximum value of thermohydraulic performance parameter has been found to be 1.8 for the range of parameters investigated. Comparisons with previously published work have been performed and found to be in excellent agreement.

## 1. Introduction

Solar energy is the most readily available source of energy. Solar air heating systems have been developed with a primary aim of collecting maximum amount of heat energy at minimum pumping cost. Solar air heater transforms solar radiation into heat and transfers that heat to air. Many types of solar heating systems have been developed by the effort to reduce the use of gas, oil, electric, and other such heat sources. One of the most potential application of solar air heater is the supply of hot air for drying of agriculture products and marine products and heating of buildings to maintain comfortable environment especially in winter season. Conventional solar air heaters have poor thermal efficiency primarily due to the low-convective heat transfer coefficient between the absorber plate and flowing air stream [1, 2]. The convective heat transfer between absorber plate and flowing air can be increased by increasing the level of turbulence by breaking the laminar viscous sublayer. The use of artificial roughness on heated surface is one of the passive techniques which is used to enhance the heat transfer. Inevitably, the enhancement in heat transfer accompanies a higher pressure drop penalty of the fluid flow. In order to keep the friction losses at a low level, the turbulence must be created only in the region very close to the duct surface, that is, in the laminar sublayer. Solar air heater with artificial roughness which is in the form of fine wires of different shapes, sizes, and orientations on the underside of the absorber plate is one of the important and effective design improvements that has been proposed to improve the thermohydraulic performance.

The performance of artificially roughened solar air heater has been tested experimentally over years for different shapes, sizes, and orientations of roughness elements to enhance the heat transfer coefficient with minimum pumping power. On the basis of literature review of the published paper, we can conclude that a lot of experimental works have been done on the performance evaluation of artificially roughened solar air heater. Detail information of different experimental works on artificially roughened solar air heater may be found in excellent review papers by Hans et al. [3], Bhushan and Singh [4], and Kumar et al. [5].

The literature search in this field also revealed that very few studies have been performed to evaluate the performance of artificially roughened solar air heater using computational fluid dynamics (CFD) approach. Due to the recent rapid growth of powerful computer resources and the development of general purpose CFD software packages, CFD can nowadays be applied to solve industrial flow problems. Today, CFD has already proven to be a valuable tool to complement experimental findings in flow structure studies. In a computational simulation, the flow structure is computed by solving the mathematical equations that govern the flow dynamics. The result is a complete description of the three-dimensional flow in the entire flow domain in terms of the velocity field and pressure distribution, including profiles of temperature variations, density, and other related physical quantities. Today's CFD codes include in their basic flow computations effects of heat and mass transfer and a range of physical and chemical models. These extensions are indispensable for application of CFD in technological process flow problems. Chaube et al. [6] conducted two dimensional CFD-based analysis of an artificially roughened solar air heater having ten different ribs shapes, namely, rectangular, square, chamfered, triangular, and so forth, provided on the absorber plate. CFD code, FLUENT 6.1 and SST *k*-*ω* turbulence model were used to simulate turbulent airflow. The best performance was found with rectangular rib of size 3 × 5 mm, and CFD simulation results were found to be in good agreement with existing experimental results. Kumar and Saini [7] performed three-dimensional CFD-based analysis of an artificially roughened solar air heater having arc shaped artificial roughness on the absorber plate. FLUENT 6.3.26 commercial CFD code and Renormalization group (RNG) *k*-*ε* turbulence model were employed to simulate the fluid flow and heat transfer. Overall enhancement ratio with a maximum value of 1.7 was obtained, and results of the simulation were successfully validated with experimental results. Karmare and Tikekar [8] carried out CFD investigation of an artificially roughened solar air heater having metal grit ribs as roughness elements on the absorber plate. Commercial CFD code FLUENT 6.2.16 and Standard *k*-*ε* turbulence were employed in the simulation. Authors reported that the absorber plate of square cross-section rib with 58° angle of attack was thermohydraulically more efficient. Gandhi and Singh [9] employed wedge-shaped ribs roughness in their simulation works. Simulation of artificially roughened solar air heater by using FLUENT showed reasonably good agreement with the experimental observations except for the friction factor. Yadav and Bhagoria [10] employed triangular-shaped rib roughness on the absorber plate to predict heat transfer behavior of an artificially roughened solar air heater by adopting CFD approach. ANSYS FLUENT 12.1 and RNG *k*-*ε* turbulence model were employed in their simulation. From 1.4 to 2.7 times enhancement in the Nusselt number was observed as compared to smooth solar air heater. Yadav and Bhagoria [11] carried out CFD investigation of an artificially roughened solar air heater having circular transverse wire rib roughness on the absorber plate. A two-dimensional CFD simulation was performed using ANSYS FLUENT 12.1 code as a solver with RNG *k*-*ε* turbulence model. The maximum value of thermal enhancement factor was reported to be 1.65 for the range of parameters investigated. A CFD-based study of conventional solar air heater was performed by Yadav and Bhagoria [12]. ANSYS FLUENT and RNG *k*-*ε* turbulence model were used to analyze the nature of the flow. Results predicted by CFD were found to be in good agreement with existing empirical correlation results. Yadav and Bhagoria [13] conducted a numerical analysis of the heat transfer and flow friction characteristics in an artificially roughened solar air heater having square-sectioned transverse ribs roughness considered to be at underside of the top heated wall. The thermohydraulic performance parameter under the same pumping power constraint was calculated in order to examine the overall effect of the relative roughness pitch. The maximum value of thermohydraulic performance parameter was found to be 1.82 corresponding to relative roughness pitch of 10.71. Yadav and Bhagoria [14] carried out a numerical investigation of turbulent flows through a solar air heater roughened with semicircular-sectioned transverse rib roughness on the absorber plate. The physical problem was represented mathematically by a set of governing equations, and the transport equations were solved using the finite element method. The numerical results showed that the flow-field, the average Nusselt number, and average friction factor are strongly dependent on the relative roughness height. The thermohydraulic performance parameter was found to be the maximum for the relative roughness height of 0.042. For more details about different CFD investigations on roughness elements of different shapes, sizes, and orientations, readers are referred to the authors' another published review paper, Yadav and Bhagoria [15].

On the basis of literature review, it is observed that very little work has been done on CFD investigation of artificially roughened solar air heater having square-sectioned transverse rib roughness on the absorber plate. The present study aims to bridge the gap in the knowledge by systematically studying the influence of the square-sectioned transverse rib roughness on heat transfer and fluid friction in a solar air heater by using a novel CFD study. This study has been performed by using commercial CFD software ANSYS FLUENT v 12.1. The main purpose of present work is to investigate the effect of relative roughness height on the average Nusselt number, average friction factor, and thermohydraulic performance parameter in an artificially roughened solar air heater having square-sectioned transverse rib roughness by adopting CFD approach.

## 2. Modelling and Numerical Simulation

A two-dimensional CFD simulation of artificially roughened solar air heater is carried out using the CFD software package ANSYS FLUENT (version 12.1) that uses the finite-volume method to solve the governing equations. The computational domain and the numerical procedure are presented in the following subsections.

### 2.1. Geometry and Mesh

**Figure 1:**
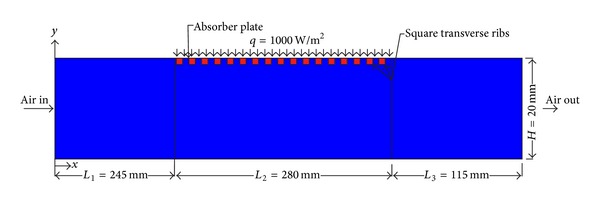
Geometry of two-dimensional computational domain.

**Figure 2:**
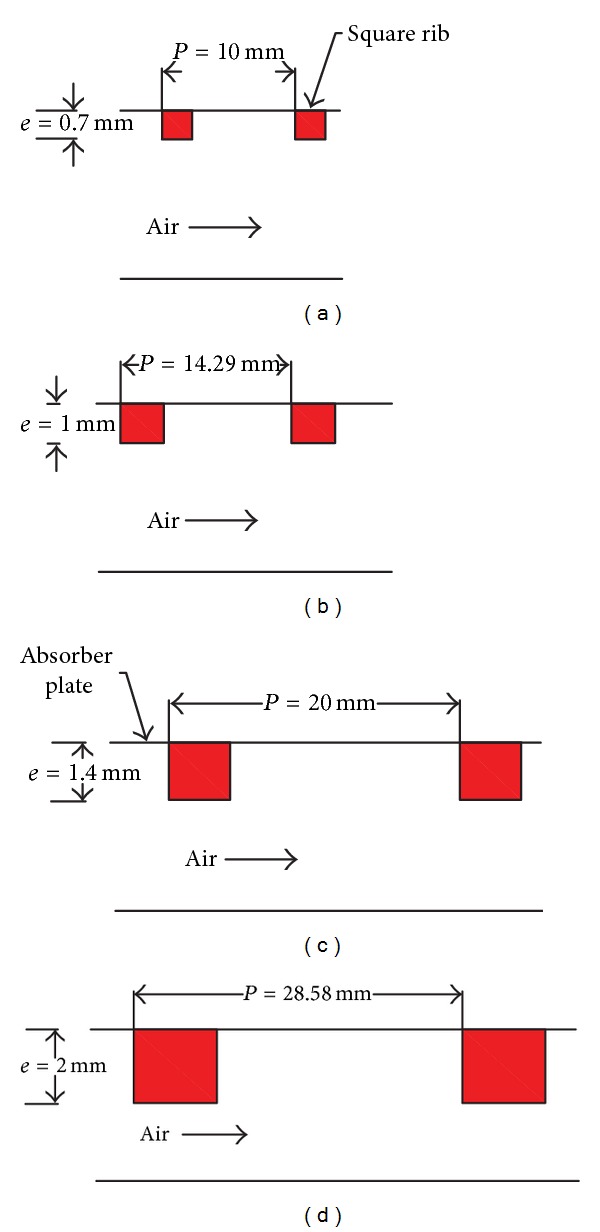
Roughened absorber plate with different configuration of square-sectioned transverse ribs.

A computational model has been created in ANSYS DESIGN MODELER v 12.1 as shown in Figure 1, which is similar to computational domain of Yadav and Bhagoria [13]. Yadav and Bhagoria [11] suggested that a suitable 2D numerical model is able to simulate well the turbulent flow and forced-convection characteristics of an artificially roughened solar air heater having circular rib on the absorber plate, such that the application of a much more complicated and expensive 3D model can be avoided. In this work, 2D computational domain is therefore chosen for saving computer memory and computational time. The solution domain has been created as per the ASHRAE Standard [16] and consisted of three sections, namely, entrance section (*L*_1_), test section (*L*_2_), and exit section (*L*_3_). The internal duct cross section is 100 × 20 mm^2^. Four different values of rib pitch (*P*) and rib height (*e*) are taken such that the relative roughness pitch remains constant. Selection of the different values of rib pitch (*P*) and rib height (*e*) based on the optimum values of these parameters was reported in the literature [3–5]. The relative roughness height, *e*/*D*, varies from 0.021 to 0.06, and the Reynolds number, Re, varies from 3800 to 18,000 (relevant in solar air heater).  Table 1 represents rib pitch (*P*), rib-height (*e*), relative roughness pitch (*P*/*e*), and relative roughness height (*e*/*D*) for different roughness configurations used in present study. Gupta et al. [17] suggested that the solar air heater systems operating in a specified range of Reynolds number (3800–18,000) show better thermohydraulic performance. The top wall consists of 0.5 mm thick absorber plate made up of aluminum. Artificial roughness in the form of square-sectioned transverse rib is considered to be at the underside of the top of the duct on the absorber plate, while other sides are considered as smooth surface. The minimum rib height, 0.7 mm, has been chosen so that the laminar sublayer is of the same order as of roughness height. The rib height, 2.0 mm, has been chosen so that the fin and flow passage blockage effects may be negligible. A uniform heat flux of 1000 W/m^2^ is considered to be on the top of the absorber plate for numerical analysis. The geometrical and operating parameters employed in this CFD investigation are listed in Table 2. A typically roughened absorber plate with different arrangement of square-sectioned transverse ribs has been shown in Figure 2.

**Figure 3:**
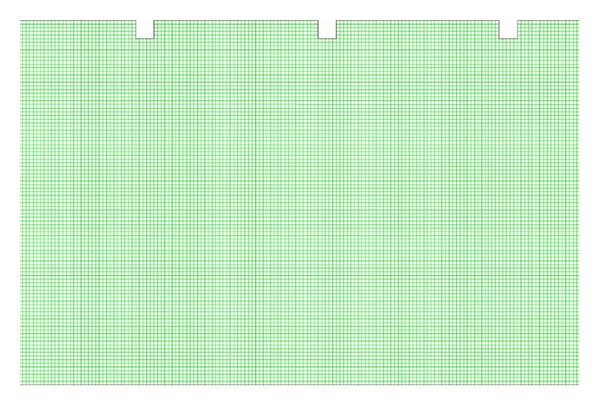
Visualization of uniform mesh distribution.

Uniform grids have been adopted for the solution of the two-dimensional governing equations for mass, momentum, and energy for all numerical simulations performed in this work. A uniform grid has been extensively utilized to accelerate mesh generation in 2D. Uniform grids are generated using ANSYS ICEM CFD v 12.1 software. A uniform grid contained 384,678 quad cells with cell size of 0.22 mm is used to resolve the laminar sublayer as shown in Figure 3. A grid independence test is carried out to determine the best mesh spacing for the geometrical mode. An extensive test for the confirmation of grid independence of the model is carried out by increasing the mesh density and adopting various mesh grading until further refinement shows a difference of less than 1% in two consecutive sets of results.

### 2.2. Governing Equations

CFD methods consist of numerical solutions of mass, momentum, and energy conservation with other equations like species transport. The solution of these equations accomplishes with numerical algorithm and methods. Two-dimensional governing equations are summarized as follows.

Continuity equation is as follows:

(1) $$\begin{array}{c} \frac{\partial }{{\partial x}_{i}}\left(\rho u_{i}\right)=0. \end{array}$$

Momentum equation is as follows:

(2) ∂∂xi(ρuiuj) =−∂p∂xi+∂∂xj[μ(∂ui∂xj+∂uj∂xi)]+∂∂xj(−ρui´uj´−).

Energy equation is as follows:

(3) $$\begin{array}{c} \frac{\partial }{{\partial x}_{i}}\left(\rho u_{i}T\right)=\frac{\partial }{\partial x_{j}}\left(\left(\Gamma +\Gamma_{t}\right)\frac{\partial T}{\partial x_{j}}\right), \end{array}$$

where Γ and Γ_*t*_ are molecular thermal diffusivity and turbulent thermal diffusivity, respectively and are given by

(4) $$\begin{array}{c} \Gamma \;=\frac{\mu }{\text{Pr}},\\ \Gamma_{t}\;=\frac{\mu_{t}}{{\text{Pr}}_{t}}. \end{array}$$

### 2.3. Turbulence Model and Boundary Conditions

There are so many different turbulence models that no single code can contain even a small subset of them. Many turbulence models, especially two-equation models, have been optimized for a particular class of flow. With all this specialization, it is hard to make blanket statements about these models. Main approach to turbulence modeling looks solely at the solutions generated using a given turbulence model and compares the solutions to those generated by others and to experimental data. According to this line of reasoning, the best turbulence model is simply the one that best matches the experimental data; no matter what its origin is.

In the present numerical simulation, Renormalization-group (RNG) *k*-*ε* model has been selected to simulate the heat transfer and fluid flow characteristics on the basis of its closer results to the Dittus-Boelter empirical correlation and the Blasius empirical correlation results. Selection of best turbulence model for the simulation of an artificially roughened solar air heater has been described clearly in the authors' another papers, Yadav and Bhagoria [11, 15]. More details of other turbulence model can be found in [18]. The modeled turbulent kinetic energy, *k*, and its rate of dissipation, *ε*, are obtained from the following transport equations for Renormalization-group (RNG) *k*-*ε* model:

(5) $$\begin{array}{c} \frac{\partial }{\partial x_{i}}\left(\rho ku_{i}\right)=\frac{\partial }{\partial x_{j}}\left(\alpha_{k}\mu_{\text{eff}}\frac{\partial k}{\partial x_{j}}\right)+G_{k}-\rho \epsilon ,\\ \frac{\partial }{\partial x_{i}}\left(\rho \epsilon u_{i}\right)=\frac{\partial }{\partial x_{j}}\left(\alpha_{\varepsilon }\mu_{\text{eff}}\frac{\partial \varepsilon }{\partial x_{j}}\right)+C_{1\varepsilon }\frac{\varepsilon }{k}\left(G_{k}\right)-C_{2\varepsilon }\rho \frac{\epsilon^{2}}{k}-R_{\varepsilon }. \end{array}$$

In these equations, *G*_*k*_ represents the generation of turbulent kinetic energy due to the mean velocity gradients; this term may be defined as

(6) $$\begin{array}{c} G_{k}=-\rho \overset{-}{\overset{´}{u_{i}}\overset{´}{u_{j}}}\frac{\partial u_{j\;}}{\partial x_{i}}, \end{array}$$

where *μ*_eff_ represents the effective turbulent viscosity and is given by

(7) $$\begin{array}{c} \mu_{\text{eff}}=\mu +\mu_{t}. \end{array}$$

The turbulent (or eddy) viscosity, *μ*_*t*_, is computed by combining *k* and *ε* as follows:

(8) $$\begin{array}{c} \mu_{t}=\rho C_{\mu }\frac{k^{2}}{\varepsilon }, \end{array}$$

where *C*_*μ*_ is a constant.

The quantities *α*_*k*_ and *α*_*ε*_ are the inverse effective turbulent Prandtl numbers for *k* and *ε*, respectively.

The model constants *C*_1*ε*_, *C*_2*ε*_, *C*_3*ε*_, *α*_*k*_, and *α*_*ε*_ have the following default values [19]:

(9) $$\begin{array}{c} C_{1\varepsilon }=1.42,\;\;C_{2\varepsilon }=1.68,\;\;C_{\mu }=0.0845,\\ \alpha_{k}=1.39,\;\;\alpha_{\varepsilon }=1.39. \end{array}$$

The governing equations are solved with the appropriate boundary conditions using ANSYS FLUENT v 12.1, a finite volume-based CFD code. The boundary conditions for the different edges can be created while constructing the geometry of the grid in ANSYS ICEM CFD V 12.1. The model has a velocity inlet on one end face and a pressure outlet on the other. A uniform air velocity (corresponding to different values of Reynolds number) is introduced at the inlet, while a pressure outlet condition with fixed pressure of 1.013 × 10^5^ Pa is applied at the exit. Constant velocity of air with 300 K is assumed in the flow direction. The temperature of air inside the duct is also taken as 300 K at the beginning. Impermeable boundary and noslip wall conditions have been implemented over the duct walls. The constant flux of 1000 W/m^2^ is given at absorber plate (top wall), while the bottom wall is kept at adiabatic wall condition. The physical properties of the air have been assumed to remain constant at mean bulk temperature. The thermophysical properties of working fluid and absorber plate are listed in Table 3.

### 2.4. Solution Method

The continuity, momentum, and energy equations in their steady, two-dimensional, turbulent, and incompressible form, along with the associated boundary conditions have been solved using the general purpose computational fluid dynamics (CFD) software, ANSYS FLUENT 12.1. Governing equations of the system are solved by finite-volume method employing semi-implicit method for pressure-linked equations (SIMPLE) algorithm. The second order upwind scheme is used for discretization of the equations [20]. In the present CFD investigation, Renormalization-group (RNG) *k*-*ε* model has been employed to simulate the flow and heat transfer. The convergence criteria for all the dependent variables are specified as 0.001. Whenever convergence problems are noticed, the solution is started using the first-order upwind discretization scheme and continued with the second-order upwind scheme. Convergence has been achieved within 1000 iterations, where the normalized residual remained constant.

## 3. Data Reduction

The main aim of present CFD work is to investigate the average Nusselt number and average friction factor in artificially roughened solar air heater having square-sectioned transverse rib roughness on the underside of the absorber plate.

Average Nusselt number for artificially roughened solar air heater is computed by

(10) $$\begin{array}{c} {\text{Nu}}_{r}=\frac{hD}{k}, \end{array}$$

where *h* is convective heat transfer coefficient.

The average friction factor for artificially roughened solar air heater is computed by

(11) $$\begin{array}{c} f_{r}=\frac{\left(\Delta P/l\right)D}{2\rho v^{2}}, \end{array}$$

where Δ*P* is pressure drop across the duct of an artificially roughened solar air heater.

It is important to note that the enhancement of heat transfer as a result of using artificial roughness is accompanied by a considerable enhancement of friction losses. This results in considerably large additional pumping costs. Consequently any enhancement scheme must be evaluated on the basis of the consideration of pumping costs. A well-known method of such evaluation is that proposed by Webb and Eckert [21] in the form of thermohydraulic performance parameter. The thermohydraulic performance parameter is defined as the ratio of the heat transfer coefficient of an augmented surface to that of a smooth surface at an equal pumping power:

(12) Thermohydraulic  performance  parameter =  (Nur/Nus)(fr/fs)1/3.

For an enhancement scheme to be viable, the value of this index must be greater than unity.

Nu_*s*_ represents Nusselt number for smooth duct of a solar air heater and can be obtained by the Dittus-Boelter equation [22].

Dittus-Boelter equation:

(13) Nus=0.023Re0.8Pr0.4,

where *f*_*s*_ represents friction factor for smooth duct of a solar air heater and can be obtained by the Blasius equation [23].

The Blasius equation is as follows:

(14) fs=0.0791Re−0.25.

## 4. Results and Discussion

### 4.1. Grid Independence Test

A grid-dependency study is carried out to evaluate mesh suitability for the turbulent flow through the artificially roughened solar air heater. A grid independence test is implemented over grids with different numbers of cells 192715, 284152, 384678, and 429413 that are used in four steps. It is found that the variation in Nusselt number and friction factor is marginal increase when moving from 384678 cells to 429413. Hence, there is no such advantage in increasing the number of cells beyond this value. Thus, the grid system of 384678 cells is adopted for the current computation.

### 4.2. Heat Transfer

**Figure 4:**
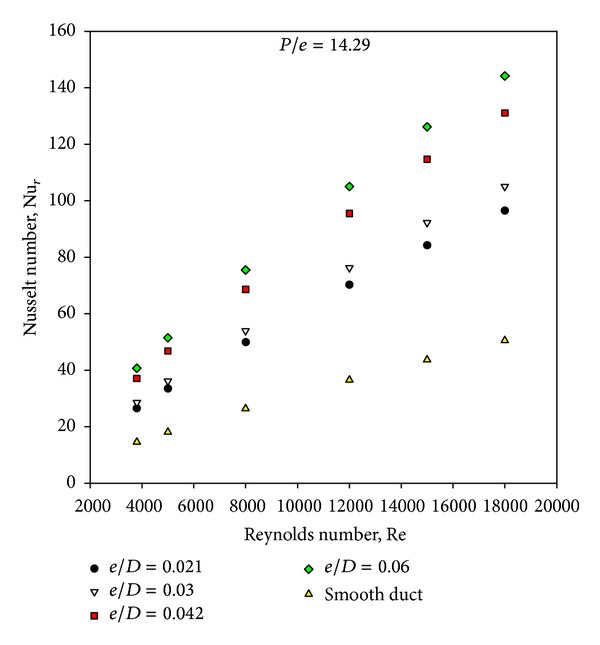
Variation of average Nusselt number with Reynolds number.

Figure 4 shows the variation of Nusselt number with Reynolds number and relative roughness height for given value of relative roughness pitch. The values of Nusselt number are found to increase with increasing value of Reynolds number in all cases as expected. The artificially roughened solar air heater can be seen to yield higher Nusselt number as compared to that of the smooth solar air heater. The vortices induced around the square ribs are responsible for the increase in the intensity of turbulence which leads to higher heat transfer rate. It is also seen that Nusselt number values increase with the increase in relative roughness height for fixed value of relative roughness pitch. The maximum value of Nusselt number occurs at a relative roughness height of 0.06 at a Reynolds number of 18,000.

**Figure 5:**
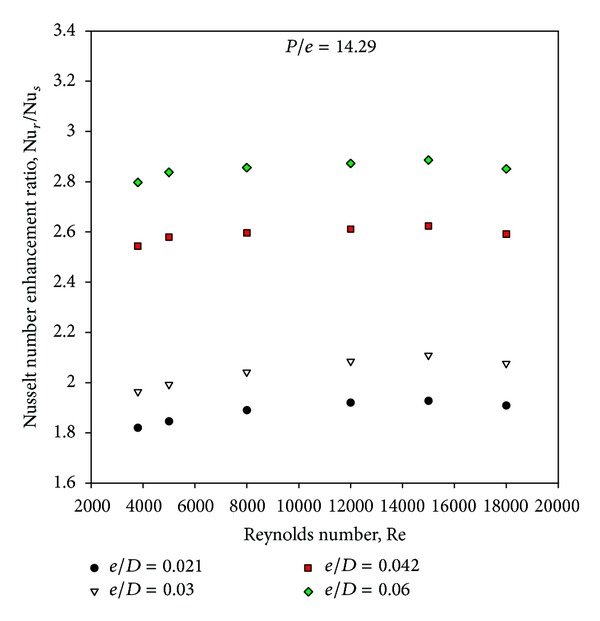
Variation of average Nusselt number enhancement ratio with Reynolds number.

Figure 5 has been drawn to depict the effect of square-sectioned transverse rib roughness on the underside of the absorber plate on Nusselt number ratio (enhancement) as a function of relative roughness height for fixed value of relative roughness pitch. It can be seen that there is a substantial enhancement caused as a result of providing artificial roughness in the form of square-sectioned transverse rib. The Nusselt number ratio enhancement achieved varies from 1.82 to 2.89 for the entire data generated from this CFD investigation. The Nusselt number ratio increases with increase in relative roughness height for all the cases. It is also seen that the Nusselt number ratio increases, attains maxima, and then decreases with an increase of Reynolds number for the selected range of parameters. The maximum enhancement in Nusselt number is found to be 2.89 times that of smooth duct corresponding to relative roughness height of 0.06 at a Reynolds number of 15,000 for the investigated range of parameters.

**Figure 6:**
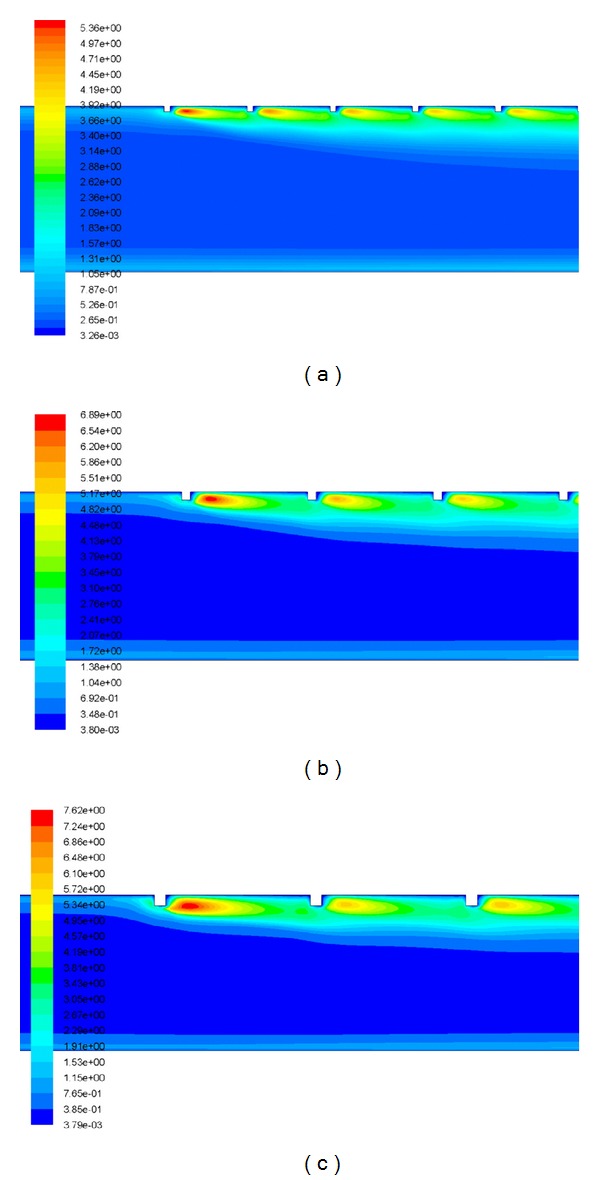
The contour plot of turbulent kinetic energy for *Re* = 18,000 and *P*/*e* = 14.29 at a relative roughness height of (a) *e*/*D* = 0.03, (b) *e*/*D* = 0.42, and (c) *e*/*D* = 0.06.

**Figure 7:**
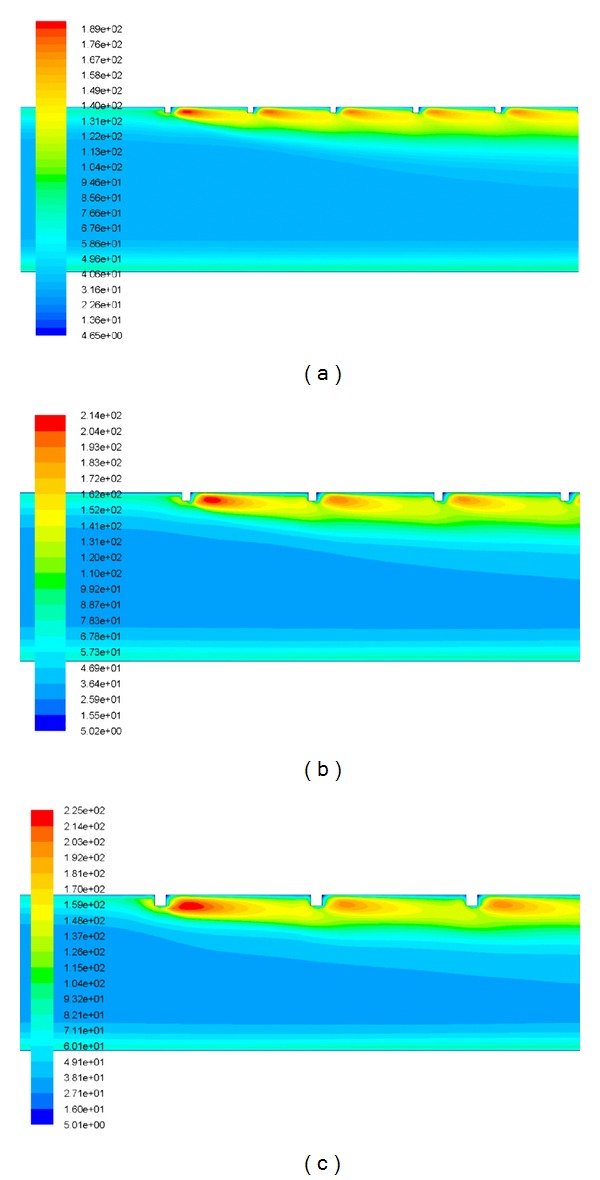
The contour plot of turbulent intensity for *Re* = 18,000 and *P*/*e* = 14.29 at a relative roughness height of (a) *e*/*D* = 0.03, (b) *e*/*D* = 0.42, and (c) *e*/*D* = 0.06.

**Figure 8:**
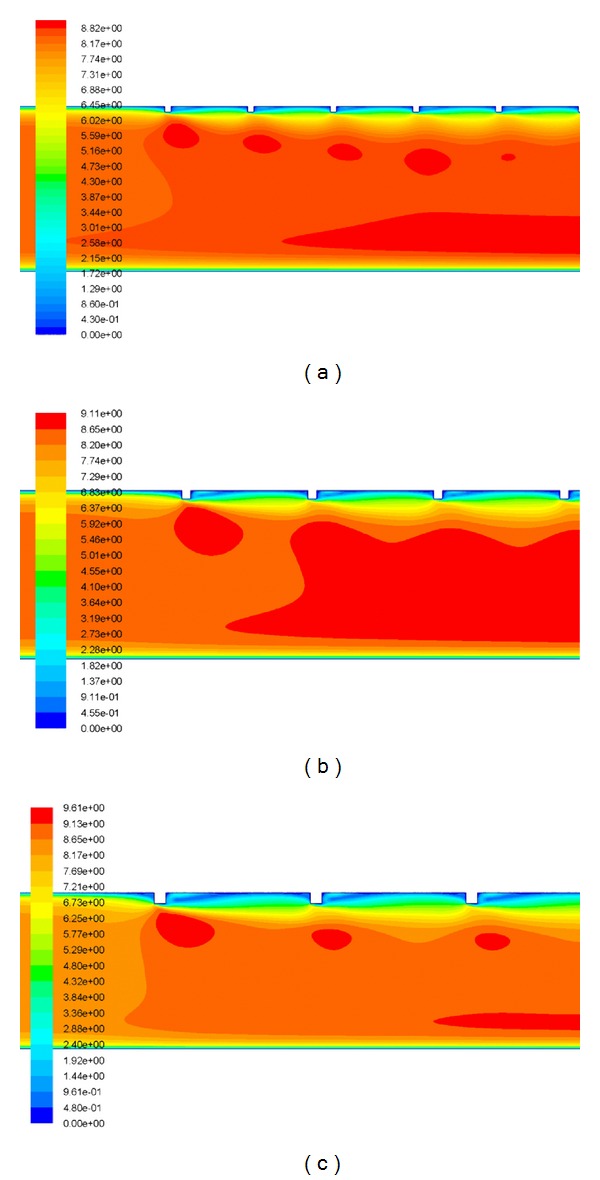
The contour plot of velocity for *Re* = 18,000 and *P*/*e* = 14.29 at a relative roughness height of (a) *e*/*D* = 0.03, (b) *e*/*D* = 0.42, and (c) *e*/*D* = 0.06.

It is well known that increase in Reynolds number increases turbulent kinetic energy and turbulent dissipation rate, which leads to the increase in the turbulent intensity and thus increases the Nusselt number. Heat transfer phenomena can be understood in a better way by the contour plot of turbulent kinetic energy.  Figure 6 shows the contour plot of turbulent kinetic energy for different values of relative roughness height at a fixed value of Reynolds number of 18,000 and relative roughness pitch of 14.29. The peak value of turbulent kinetic energy is occurred near the top-heated wall on the downstream side of the rib, and then it decreases with the increase in distance from the wall. Further, heat transfer phenomena can also be analyzed and described by contour plot of turbulent intensity.  Figure 7 shows the contour plot of turbulent intensity for different values of relative roughness height at a fixed value of Reynolds number of 18,000 and relative roughness pitch of 14.29. The peak value of turbulent intensity is occurred near the top-heated wall on the downstream side of the rib, and then it decreases with the increase in distance from the wall. As the Reynolds number increases, the roughness elements begin to project beyond the laminar sublayer. Laminar Sub-layer thickness decreases with an increase in the Reynolds number. In addition to this, there is local contribution to the heat removal by the vortices originating from the roughness. This increases the heat transfer rate as compared to the smooth surface.  Figure 8 shows the contour plot of velocity for different values of relative roughness height at a fixed value of Reynolds number of 18,000 and relative roughness pitch of 14.29. Along the roughened duct of a solar air heater, it can be observed that the velocity at inlet is lower than that at the outlet of the duct, due to the flow acceleration in the stream-wise direction. The instantaneous velocity contours are very irregular because of the presence of square-sectioned transverse ribs, that is, nature of turbulence.

### 4.3. Friction Factor

**Figure 9:**
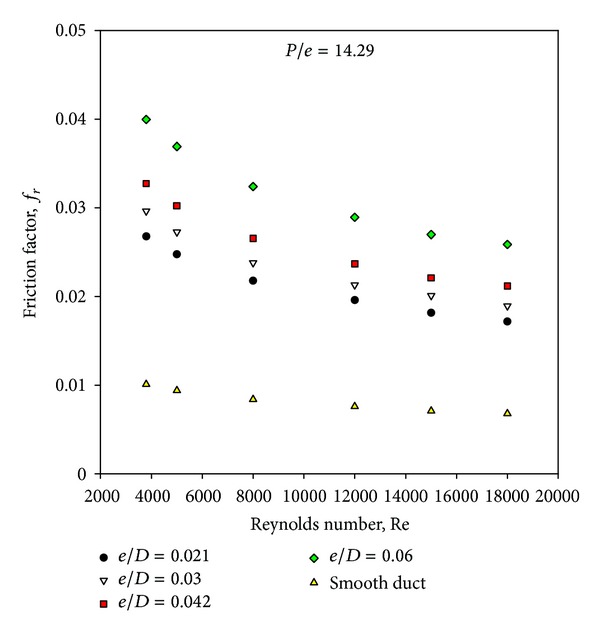
Variation of average friction factor with Reynolds number.

Figure 9 shows the variation of friction factor with Reynolds number and relative roughness height for given value of relative roughness pitch. The values of friction factor are found to decrease with increasing Reynolds number in all cases as expected due to the suppression of viscous sublayer with increase in Reynolds number. The artificially roughened solar air heater can be seen to yield higher friction factor as compared to that of the smooth solar air heater. It is also seen that friction factor values increase with the increase in relative roughness height for fixed value of relative roughness pitch. The maximum value of friction factor occurs at a relative roughness height of 0.06 at a Reynolds number of 3800.

**Figure 10:**
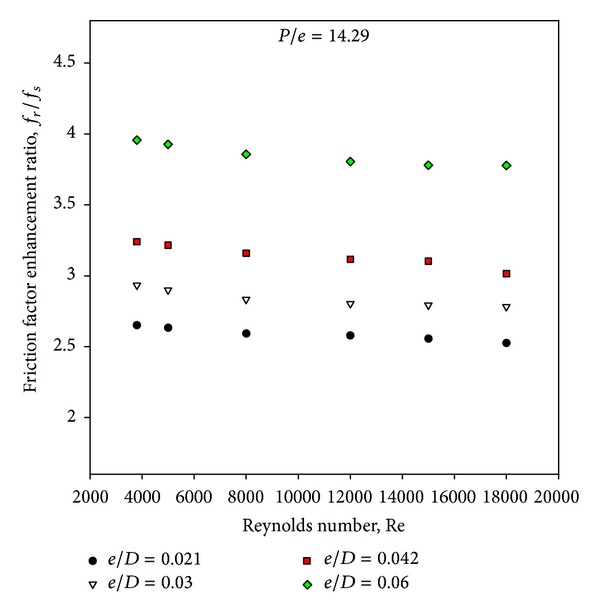
Variation of average friction factor enhancement ratio with Reynolds number.

**Figure 11:**
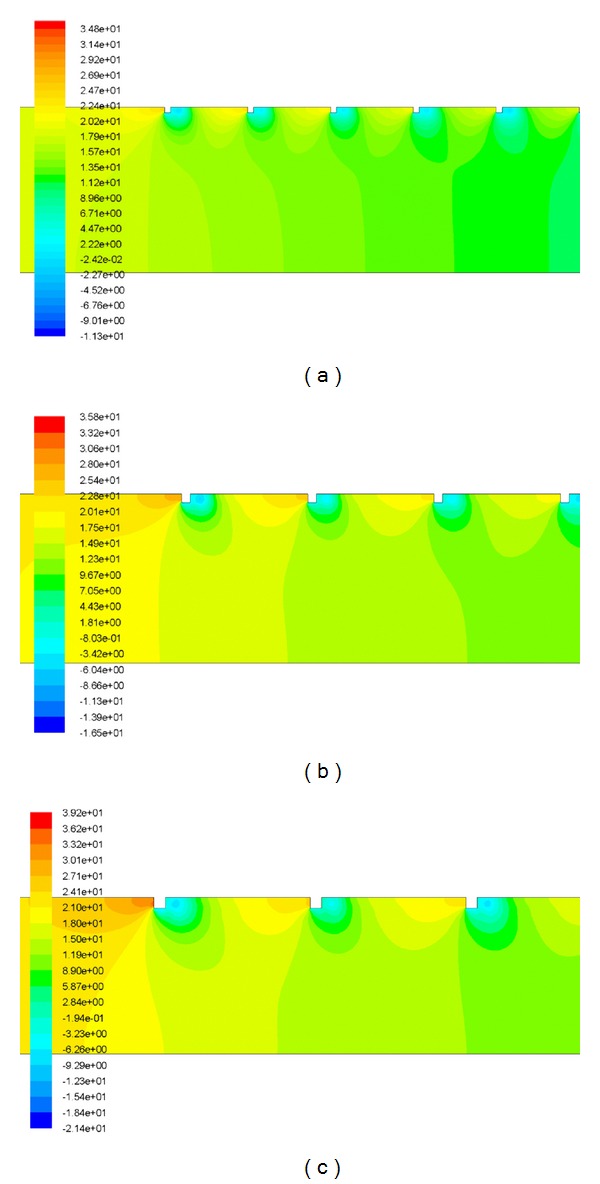
The contour plot of pressure for *Re* = 18,000 and *P*/*e* = 14.29 at a relative roughness height of (a) *e*/*D* = 0.03, (b) *e*/*D* = 0.042 and (c) *e*/*D* = 0.06.

Figure 10 has been drawn to depict the effect of square-sectioned transverse rib roughness on the underside of the absorber plate on friction factor ratio (enhancement) as a function of relative roughness height for fixed value of relative roughness pitch. It can be seen that there is a substantial enhancement caused as a result of providing artificial roughness in the form of square-sectioned transverse rib. The friction factor ratio enhancement achieved varies from 2.53 to 3.96 for the entire data generated from this CFD investigation. The friction factor ratio increases with the increase in relative roughness height for all the cases. It is also seen that the friction factor ratio decreases with an increase of Reynolds number for the investigated range of parameters. The maximum enhancement in friction factor is found to be 3.96 times that of smooth duct corresponding to relative roughness height of 0.06 at a Reynolds number of 3800 for the investigated range of parameters. The shedding of vortices originating from the square-sectioned rib top causes an additional loss of energy resulting in increased friction factor. It is also observed that the friction factor decreases with the increase in Reynolds number because of the suppression of viscous sublayer. It is found that the pressure drop is substantially increased by the presence of surface roughness over the entire range of Reynolds numbers studied (3800 < Re < 18,000).  Figure 11 shows the contour plot of pressure for different values of relative roughness height at a fixed value of Reynolds number of 18,000 and relative roughness pitch of 14.29.

### 4.4. Thermohydraulic Performance Parameter

**Figure 12:**
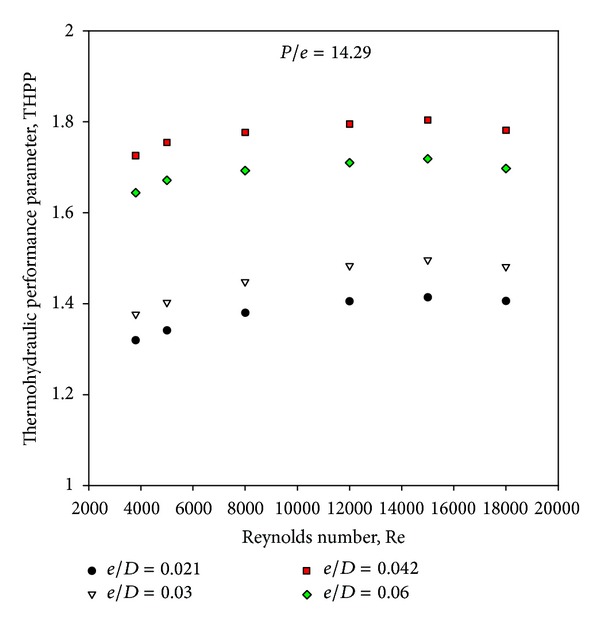
Variation of thermohydraulic performance parameters with Reynolds number.

Figures 5 and 10 show the Nusselt number ratio and friction factor ratio for different values of relative roughness height at a constant value of relative roughness pitch. Increasing the relative roughness height will increase the friction factor and Nusselt number. It is interesting to note that the rate of increase of friction factor is higher than that of the Nusselt number. This appears due to the fact that at higher values of relative roughness height, the reattachment of free shear layer might not occur, and the rate of heat transfer enhancement will not be proportional to that of friction factor. If value of relative roughness height is increased beyond a certain limit, it will cause a rapid enhancement in the friction factor than that of Nusselt number, and square-sectioned ribs will act as fins. Hence, it is essential to determine an optimum value of the relative roughness height that will result in maximum enhancement in heat transfer with minimum friction power penalty. A well-known method of such evaluation is that proposed by Webb and Eckert [21] in the form of thermohydraulic performance parameter.  Figure 12 shows the variation of the thermohydraulic performance parameter with Reynolds number for different values of relative roughness height and for fixed value of relative roughness pitch of 14.29. It can be seen that the thermohydraulic performance parameters are generally above unity for all the cases. The thermohydraulic performance parameter is seen to increase with the increase in relative roughness height up to 0.042 and then decreases with further increase in the relative roughness height at all values of the Reynolds number, thus attaining a maxima at a relative roughness height of 0.042. It is also observed that the thermohydraulic performance parameter first tends to increase then decreases with the rise of Reynolds number, attaining a maxima at a Reynolds number of 15,000. The thermohydraulic performance parameter values vary between 1.32 and 1.8 for the range of parameters investigated. Finally, the thermohydraulic performance parameter of *e*/*D* = 0.042 is found to be the best for the investigated range of parameters and is about 1.8 at a Reynolds number of 15,000.

### 4.5. Validation of Results

**Figure 13:**
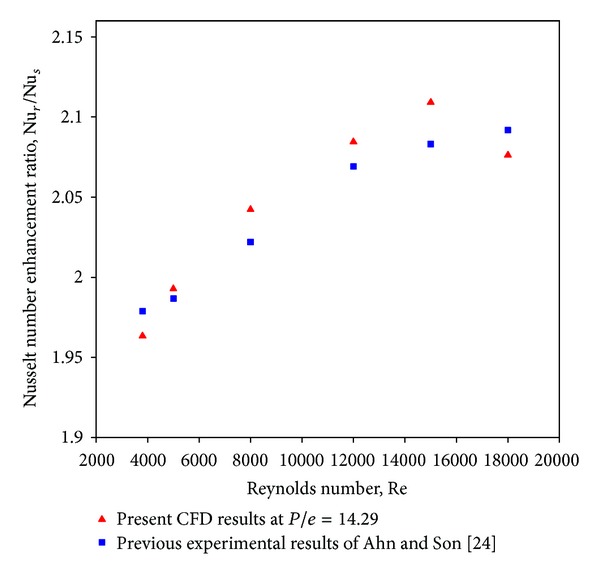
Comparison of friction factor predicted by present CFD investigation with the previous experimental results of Ahn and Son [24].

The CFD simulation result of the artificially roughened solar air heater roughened with square-sectioned transverse rib roughness has been validated with the experimental data as shown in Figure 13. In order to validate the model, it is compared with the previous experimental results of Ahn and Son [24]. Figure 13 shows the comparison of Nusselt number ratio (enhancement) predicted by present CFD investigation with the previous experimental results of Ahn and Son [24]. It can be seen that there is a good agreement between the results predicted by the present CFD investigation and experimental results of Ahn and Son [24]. The discrepancy between the experimental data and the present computational results is less than ±10%. In order to make results more reliable, the trends of average Nusselt number and average friction factor have been compared with available experimental work. Similar trends of results of average Nusselt number and average friction factor were also obtained by Chandra et al. [25] who investigated the effect of roughness types on friction factors and heat transfer in roughened rectangular duct. It can be seen that there is a good agreement between the results predicted by the present CFD investigation and previous experimental results.

## 5. Conclusions

Two-dimensional CFD analysis of a solar air heater roughened with square-sectioned transverse rib roughness on the absorber plate has been performed for four different configurations of rib roughness and six different values of Reynolds number, ranging from 3800 to 18,000. Turbulent kinetic energy, turbulent intensity, and pressure contour maps are presented for characteristic flow behaviors in artificially roughened solar air heater. In view of the present CFD predictions the following relevant conclusions are drawn.

1. Roughness height and pitch strongly affect the flow pattern and hence the performance of an artificially roughened solar air heater. The square-sectioned transverse rib roughness on the absorber plate shows appreciable heat transfer enhancement.
2. The maximum Nusselt number ratio is found to be 2.89 corresponding to relative roughness height of 0.06 at a Reynolds number of 15,000 for the investigated range of parameters.
3. The maximum friction factor ratio is found to be 3.96 corresponding to relative roughness height of 0.06 at a Reynolds number of 3800 for the investigated range of parameters.
4. It is found that the solar air heater roughened with square-sectioned transverse rib roughness on the absorber plate with *e*/*D* = 0.042 provides the better thermohydraulic performance parameter of 1.8 at a Reynolds number of 15,000 and hence can be employed for heat transfer augmentation.
5. The CFD simulation results have been validated with the previous experimental data. It can be seen that there is a good agreement between the results predicted by the present CFD investigation and previous experimental results. It can therefore be concluded that the present numerical results demonstrated the validity of the proposed system.

## Figures and Tables

**Table 1:** Configurations of artificial roughness used in the study.

Roughness configurations	Rib height, *e* (mm)	Rib pitch, *P* (mm)	Hydraulic diameter of duct, *D* (mm)	Relative roughness pitch, *P*/*e *	Relative roughness height, *e*/*D *
Type 1	0.7	10	33.33	14.29	0.021
Type 2	1	14.29			0.03
Type 3	1.4	20			0.042
Type 4	2.0	28.58			0.06

**Table 2:** Range of geometrical and operating parameters for CFD analysis.

Geometrical and operating parameters	Range
Entrance length of duct, “*L*_1_”	245 mm
Test length of duct, “*L*_2_”	280 mm
Exit length of duct, “*L*_3_”	115 mm
Width of duct, “*W*”	100 mm
Depth of duct, “*H*”	20 mm
Hydraulic diameter of duct, “*D*”	33.33 mm
Duct aspect ratio, “*W*/*H*”	5
Rib height, “*e*”	0.7, 1.0, 1.4 and 2.0 mm
Rib Pitch, “*P*”	10, 14.29, 20 and 28.58 mm
Reynolds number, “Re”	3800–18,000 (6 values)
Prandtl number, “Pr”	0.7441
Relative roughness pitch, “*P*/*e*”	14.29 (fixed value)
Relative roughness height, “*e*/*D*”	0.021, 0.03, 0.042 and 0.06

**Table 3:** Thermophysical properties of air and absorber plate for CFD analysis.

Properties	Air	Absorber plate (aluminum)
Density, “*ρ*” (kg m^−3^)	1.225	2719
Specific heat, “*C*_*p*_” (J kg^−1^ K^−1^)	1006.43	871
Viscosity, “*μ*” (N m^−2^)	1.7894*e* − 05	—
Thermal conductivity, “*k*” (W m^−1^ K^−1^)	0.0242	202.4

## Nomenclature

*C*_*p*_: Specific heat of air, J/kg K
*D*: Equivalent or hydraulic diameter of duct, mm
*e*: Rib height, mm
*e*/*D*: Relative roughness height
*f*: Friction factor
*H*: Depth of duct, mm
*h*: Heat transfer coefficient, W/m^2^ K
*k*: Thermal conductivity of air, W/m K
*L*: Length of duct, mm
*L*_1_: Inlet length of duct, mm
*L*_2_: Test length of duct, mm
*L*_3_: Outlet length of duct, mm
Nu: Nusselt number
*P*: Pitch, mm
*P*/*e*: Relative roughness pitch
Pr: Prandtl number
*Re*: Reynolds number
*v*: Velocity of air in the duct, m/s
*W*: Width of duct, mm
*W*/*H*: Duct aspect ratio
*α*: Angle of attack, degree
Γ: Molecular thermal diffusivity, m^2^/s
Γ_*t*_: Turbulent thermal diffusivity, m^2^/s
*δ*: Transition sublayer thickness, mm
Δ*P*: Pressure drop, Pa
*ε*: Dissipation rate, m^2^/s^3^
*κ*: Turbulent kinetic energy, m^2^/s^2^
*μ*: Dynamic viscosity, N s/m^2^
*μ*_*t*_: Turbulent viscosity, N s/m^2^
*ρ*: Density of air, kg/m^3^
*ω*: Specific dissipation rate, 1/s.

### Subscripts

*r*: Roughened
*s*: Smooth.
